# Supplementary material for: A recent evaluation on the performance of LLMs on radiation oncology physics using questions of randomly shuffled options
Source: Front Oncol. 2025 May 23;15:1557064. doi: 10.3389/fonc.2025.1557064 (PMC12141255; doi:10.3389/fonc.2025.1557064)
Supplement: Supplementary file 1 [file Image1.pdf]

Supplementary Materials

1 Distribution of correct answer options after random shuffling

To verify the randomness of the correct answer location, we plotted the distribution of correct answers for five new shuffled exam sets. In Fig. 1, each color represents a distinct position of the correct answer. From the figure, we can see that the locations are indeed fairly random, which supports our testing procedures with these new exam sets.

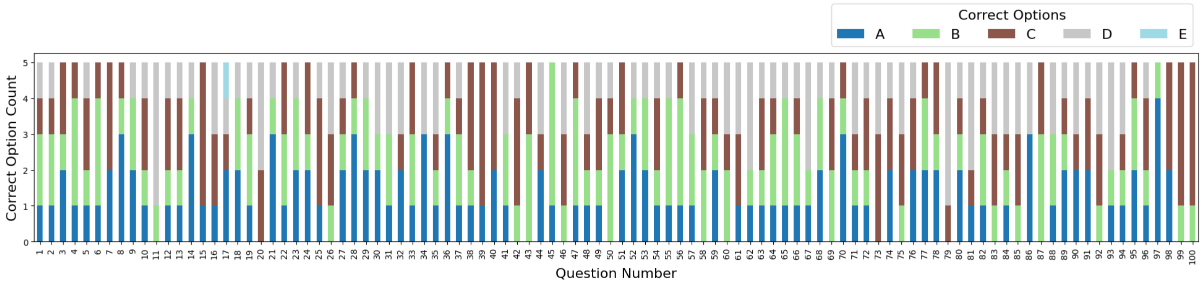

Figure 1: Distribution of correct answer options after random shuffling (note: only two questions offered option E).
